# Prognostic Value of the Modified Glasgow Prognostic Score in Patients Undergoing Radical Surgery for Hepatocellular Carcinoma

**DOI:** 10.1097/MD.0000000000001486

**Published:** 2015-09-11

**Authors:** Xiao-Chun Ni, Yong Yi, Yi-Peng Fu, Hong-Wei He, Xiao-Yan Cai, Jia-Xing Wang, Jian Zhou, Yun-Feng Cheng, Jian-Jun Jin, Jia Fan, Shuang-Jian Qiu

**Affiliations:** From the Liver Cancer Institute, Zhongshan Hospital (X-CN, YY, Y-PF, H-WH, X-YC, J-XW, JZ, JF, S-JQ); Shanghai Medical School, Fudan University (X-CN, YY, Y-PF, H-WH, X-YC, J-XW, JZ, JF, S-JQ); Key Laboratory for Carcinogenesis and Cancer Invasion, The Chinese Ministry of Education, Shanghai, People's Republic of China (X-CN, YY, Y-PF, H-WH, X-YC, J-XW, JZ, JF, S-JQ); and Biomedical Research Center, Zhongshan Hospital, Fudan University, Shanghai, 200032, People's Republic of China (Y-FC, J-JJ, S-JQ).

## Abstract

There is increasing and consistent evidence concerning the association of systemic inflammation and poor outcome in patients with hepatocellular carcinoma (HCC). The aim of this study was to identify a superior inflammation-based prognostic scoring system for patients with HCC undergoing hepatectomy.

We analyzed two independent cohorts of a total of 723 patients with HCC who underwent radical surgery between 2010 and 2012. The prognostic value of the inflammation scores, including the Glasgow Prognostic Score (GPS), modified GPS (mGPS), neutrophil-to-lymphocyte ratio, platelet lymphocyte ratio, prognostic index, and prognostic nutritional index, as well as the Barcelona Clinic Liver Cancer and Cancer of the Liver Italian Program staging systems was analyzed in a test cohort of 367 patients and validated in a validation cohort of 356 patients.

A high score with the mGPS was associated with large tumor size, vascular invasion, and advanced clinical stage. Multivariate analysis showed that the mGPS was independently associated with overall survival and disease-free survival, and had a higher area under the curve value in comparison with other inflammation-based scores.

The results of this study demonstrated that the mGPS is an independent marker of poor prognosis in patients with resectable HCC and is superior to other inflammation-based scores.

## Abstract 2

Supplemental Digital Content is available in the text

## INTRODUCTION

Hepatocellular carcinoma (HCC) is one of the most common types of malignancy worldwide and the third leading cause of cancer-related death. In contrast to other malignancies, survival of HCC patients is peculiarly influenced by the underlying liver function along with the extent of spread of the primary tumor.^1^ Several criteria have been proposed to predict patient prognosis, including functional liver reserve, performance status, as well as tumor stage; however, despite there being 7 different prognostic scales, such as the Barcelona Clinic Liver Cancer (BCLC) and Cancer of the Liver Italian Program (CLIP) staging scores, there is little consensus on which is the most reliable system for staging and predicting the prognosis of patients with HCC.^2,3^ Furthermore, many of these scores are cumbersome and rarely used in routine clinical practice. Therefore, there is an urgent need for the development of a reliable, simple, and easy-to-use prognostic score.

Numerous data have confirmed that inflammation is a critical component of tumor progression.^4,5^ The presence of a systemic inflammatory response, as indicated by an elevation in circulating C-reactive protein (CRP) levels, has been shown to be a reliable predictor of survival in patients with a variety of solid tumors, including HCC.^6,7^ The measurement of the systemic inflammatory response has been subsequently refined using a selective combination of CRP and albumin measurements (termed the Glasgow Prognostic Score, GPS) and has been shown to effectively predict the prognosis of patients with various advanced cancers.^8–10^ More recently, the results of a large cohort study showed that the modified GPS (mGPS) is a powerful prognostic factor of survival across all tumor sites studied and is independent of age, sex, and deprivation.^11,12^ Moreover, several studies have shown that some other inflammation-based prognostic scores, including the neutrophil-to-lymphocyte ratio (NLR), platelet lymphocyte ratio (PLR), prognostic index (PI), and prognostic nutritional index (PNI), have prognostic value in a variety of cancers.^13–18^

HCC is a type of tumor that slowly unfolds on a background of chronic inflammation, which is triggered by exposure to infectious agents, such as hepatitis viruses. Most of these studies have clarified that inflammation-based prognostic scores were associated with overall survival (OS) of HCC patients.^19,20^ Only a few studies have demonstrated that elevated NLR increases the risk of recurrence in patients following liver transplantation.^21–23^ Although surgical resection and liver transplantation provide valid approaches to treat HCC, long-term outcomes of patients with HCC remain unsatisfactory because of the high incidence of recurrence, as the 5-year recurrence rate after curative resection remains high at up to 54.1% to 61.5%. Therefore, it is of considerable interest to explore the relationship between inflammation-based scores and recurrence in patients with resectable HCC.

Relatively few studies have focused on the comparison of various inflammation-based prognostic scores, especially in patients who underwent surgery. Therefore, the aims of the current study were to validate the prognostic power of inflammation-based prognostic scores (the GPS, mGPS, NLR, PLR, PI, and PNI) in patients with HCC undergoing curative resection as an initial treatment and to compare the performance of these scores with established clinical prognostic models, including the BCLC stage and CLIP scores, and to ascertain whether systemic inflammation is an accurate marker of prognosis.

## METHODS

We retrospectively analyzed the records of patients who underwent radical surgery for primary HCC in the Liver Cancer Institute, Zhongshan Hospital, Fudan University (Shanghai, China), between December 2010 and January 2012. Patients demonstrating clinical evidence of infection or other inflammatory conditions or who received prior intervention or died within 30 days after surgery were excluded from this study. A total of 723 patients with HCC were finally included and evaluated. Then, we randomly divided the patients into 2 groups using a digital random table, the test cohort and validation cohort (n = 367 and 356 patients, respectively). The study protocol was approved by the Ethics Committee of Zhongshan Hospital, which is affiliated with Fudan University, and each patient provided informed consent to participate in the study.

The GPS, mGPS, NLR, PLR, PI, and PNI were constructed as described in Table 1. The baseline characteristics of the patients are summarized in Supplementary Table S1, http://links.lww.com/MD/A406. Follow-up procedures are described in our previous study.^24^ OS was defined as the interval between surgery and time of either death or last follow-up. Disease-free survival (DFS) was defined as the interval between surgery and time of recurrence. The last follow-up date for all surviving patients was July 2013. The median follow-up time was 24 months (range, 3–32 months).

**TABLE 1:**
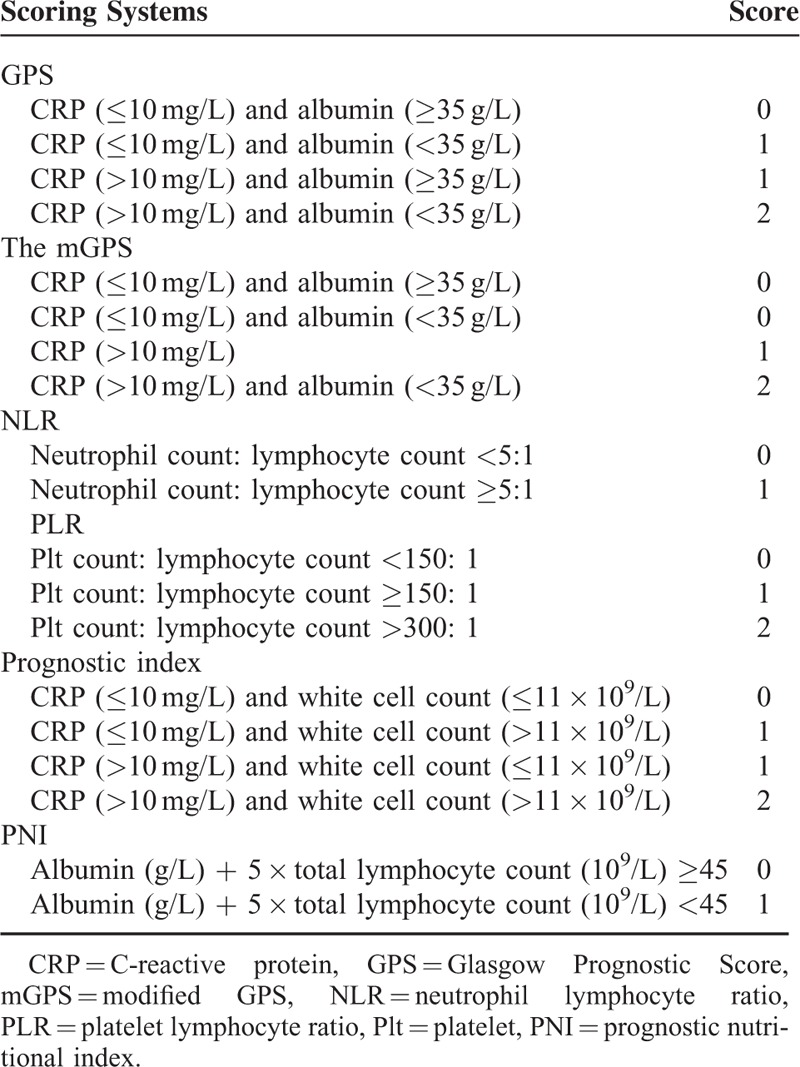
Inflammation-Based Prognostic Scores

### Statistical Analysis

All data analysis was performed using SPSS statistical software (version 16; SPSS Inc., Chicago, IL). Differences between two independent samples were tested using the Mann–Whitney *U* test (nonparametric). The Pearson chi-square test was used to identify associations between variables. Univariate and multivariate analyses were performed to assess prognostic factors using the Cox proportional hazard model. To avoid collinearity bias, the inflammation-based prognostic scores were preliminarily tested using a multivariate model that included the individual variables composing the BCLC stage and CLIP score. A receiver operating characteristics curve was also generated and the area under the curve (AUC) was calculated to evaluate the discriminatory ability of each scoring system.

## RESULTS

### Patient Demographics and Clinicopathologic Characteristics

There were no significant differences in baseline characteristics between the test and validation cohorts (Supplementary Table S1, http://links.lww.com/MD/A406). Correlations between inflammatory scores and clinicopathologic parameters of HCC patients in the test cohort are summarized in Table 2. The GPS, mGPS, and PI had a close association with alpha-fetoprotein (AFP) levels, tumor size, and vascular invasion; however, there were no significant differences in the associations between inflammatory scores and the clinical background factors of age, sex, alanine transaminase, total serum bilirubin, and gamma-glutamyl transpeptidase (GGT), although PNI was associated with age. In addition, no inflammatory scores were associated with the presence of a tumor capsule, tumor number, or Edmondson grade. There were significant associations between inflammatory scores, including the GPS, mGPS, and PI, and clinical stages (CLIP score and BCLC stage) (Table 2).

**TABLE 2:**
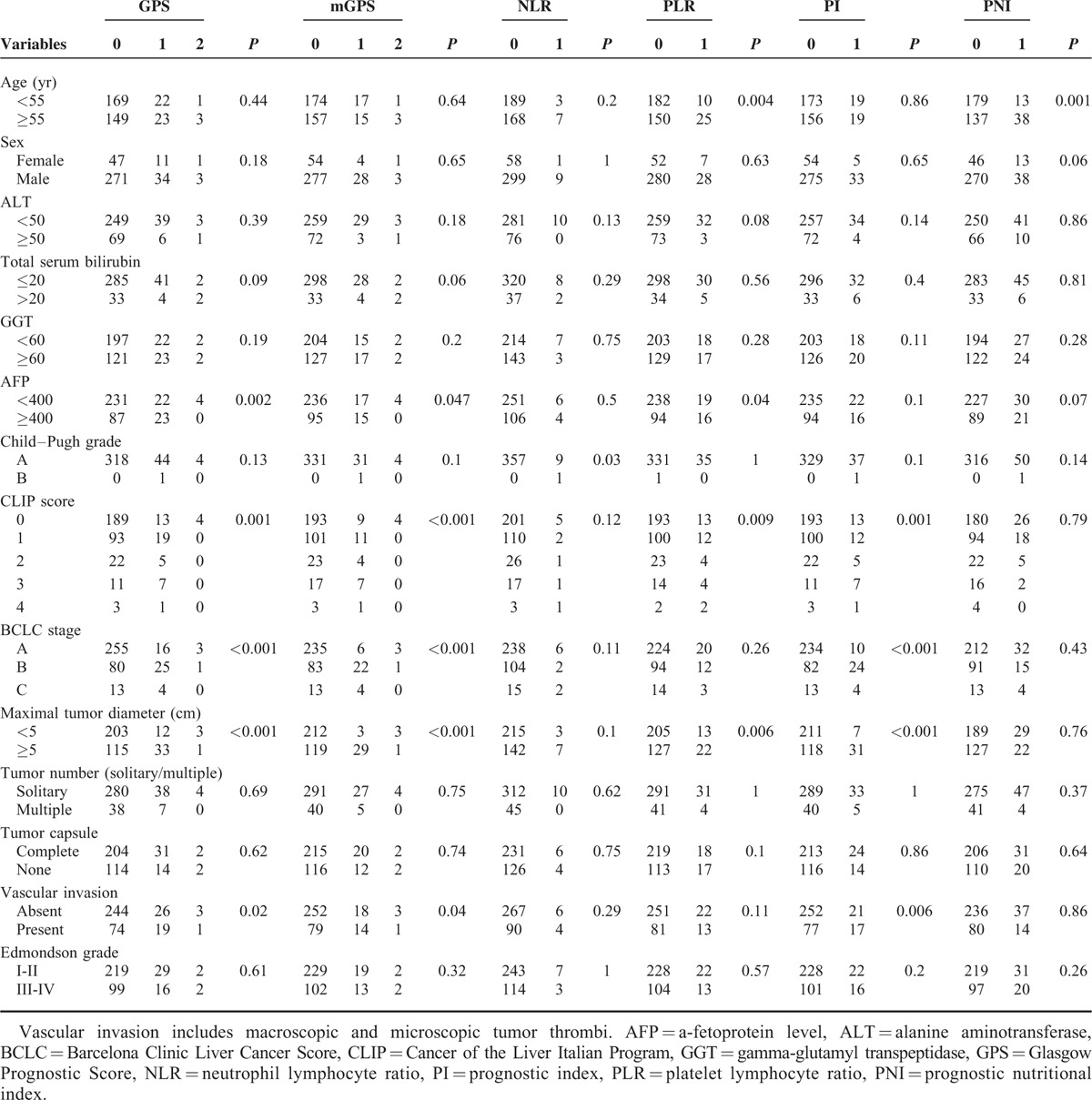
Relationship between Inflammatory Prognostic Scores and Clinical Variables in Test Cohort

### Prediction of Tumor Recurrence and Patient Survival in the Test Cohort

**FIGURE 1:**
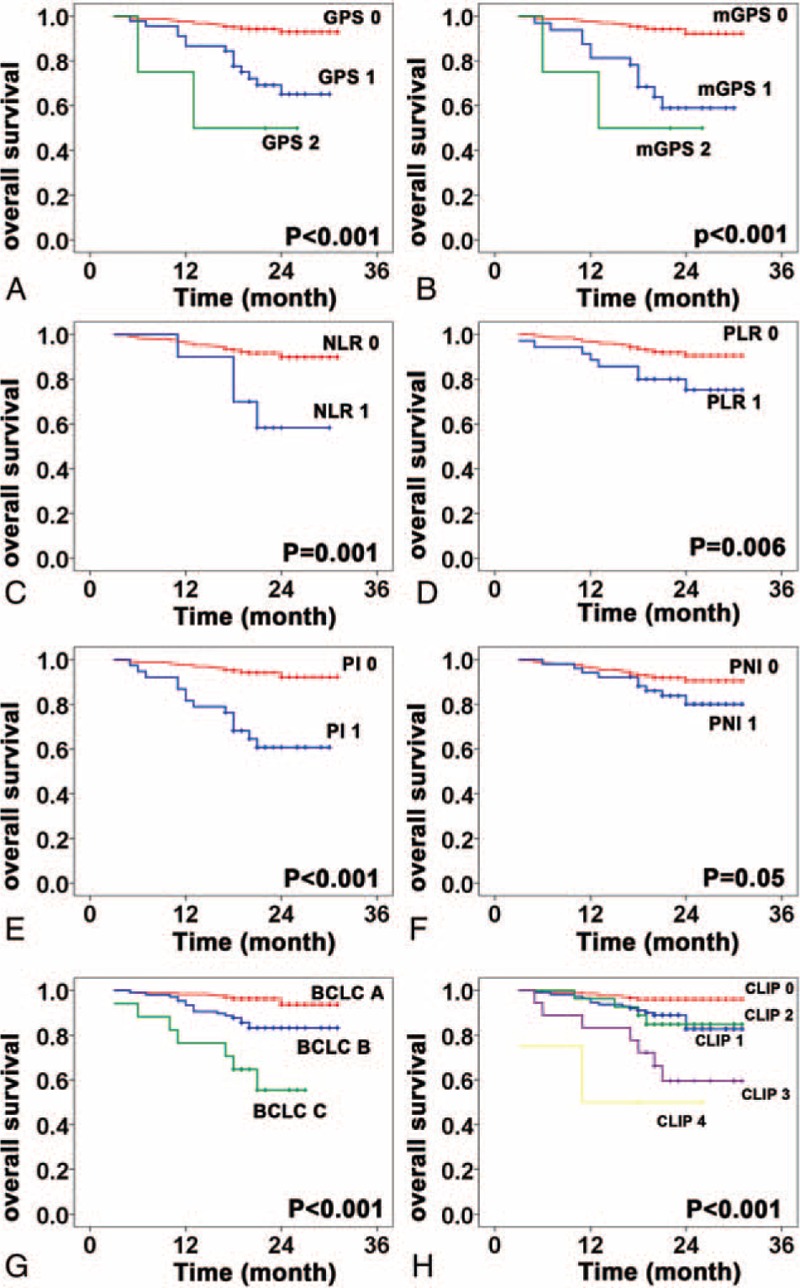
Kaplan–Meier overall survival curves for patients in the test set stratified by inflammation-based prognostic scores and stating system. A, Glasgow Prognostic Score; B, modified Glasgow Prognostic Score; C, neutrophil-to-lymphocyte ratio; D, platelet lymphocyte ratio; E, prognostic index; F, prognostic nutritional index; G, Barcelona Clinic Liver Cancer; H, Cancer of the Liver Italian Program.

**FIGURE 2:**
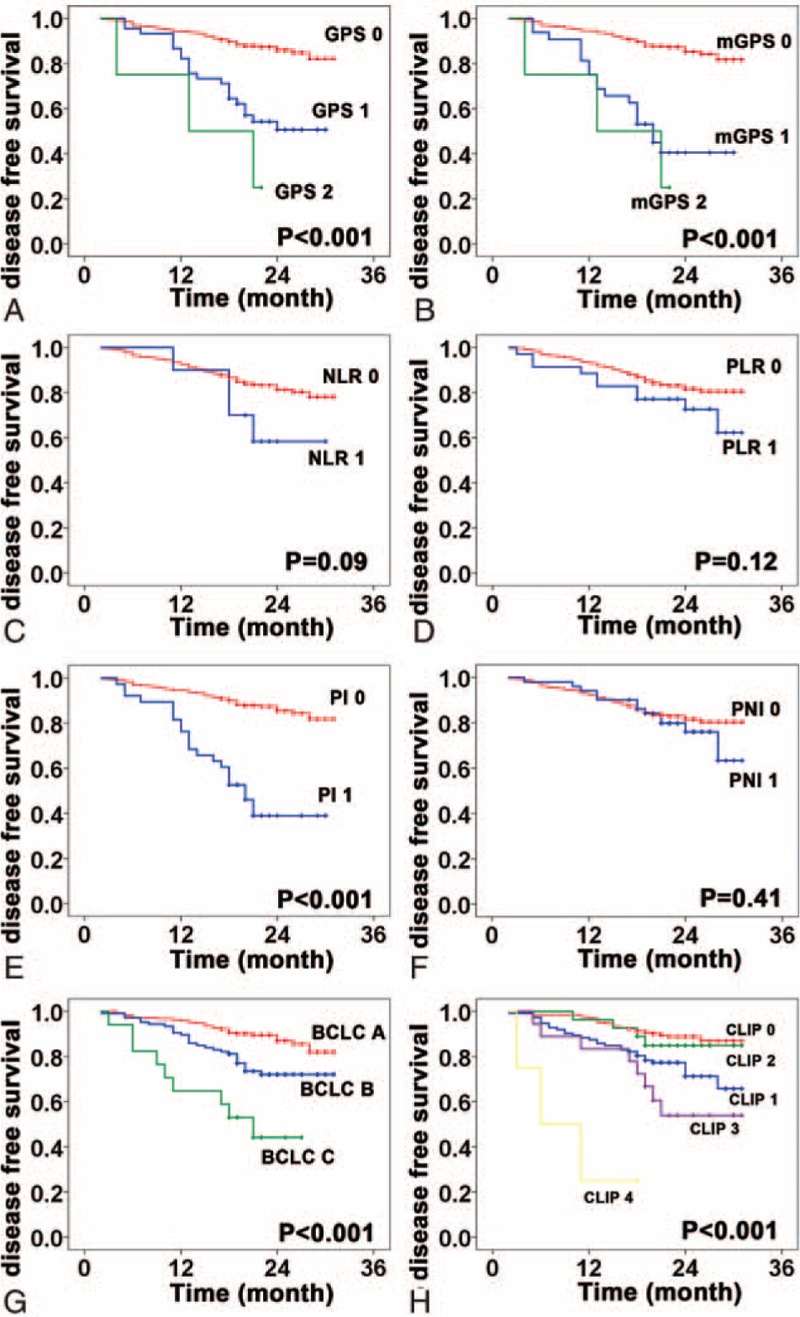
Kaplan–Meier disease-free survival curves for patients in the test set stratified by inflammation-based prognostic scores and stating system. A, Glasgow Prognostic Score; B, modified Glasgow Prognostic Score; C, neutrophil-to-lymphocyte ratio; D, platelet lymphocyte ratio; E, prognostic index; F, prognostic nutritional index; G, Barcelona Clinic Liver Cancer; H, Cancer of the Liver Italian Program.

Correlations between the inflammation-based prognostic scores and OS are shown in Figure 1. An elevated GPS, mGPS, NLR, PLR, and PI as well as BCLC stage (*P* < 0.001) and CLIP score (*P* < 0.001) were associated with reduced OS (all, *P* < 0.05). Furthermore, the GPS, mGPS, PI, BCLC stage, and CLIP score were associated with DFS (all, *P* < 0.001) (Figure 2); however, PNI was not associated with OS or DFS. Univariate analysis showed that besides the inflammation-based prognostic scores, the presence of GGT, AFP, maximal tumor diameter, and vascular invasion were significant predictors of OS and DFS. Multivariate analysis confirmed that the mGPS, AFP, GGT, and vascular invasion were independent predictors of both OS and DFS. Then, a multivariate Cox regression model was used to further assess the independent prognostic ability of BCLC stage and CLIP score. The analysis showed that the mGPS, GGT, and CLIP score were significant prognostic factors for OS, whereas the mGPS, GGT, AFP, and BCLC stage were significant prognostic factors for DFS (Table 3).

**TABLE 3:**
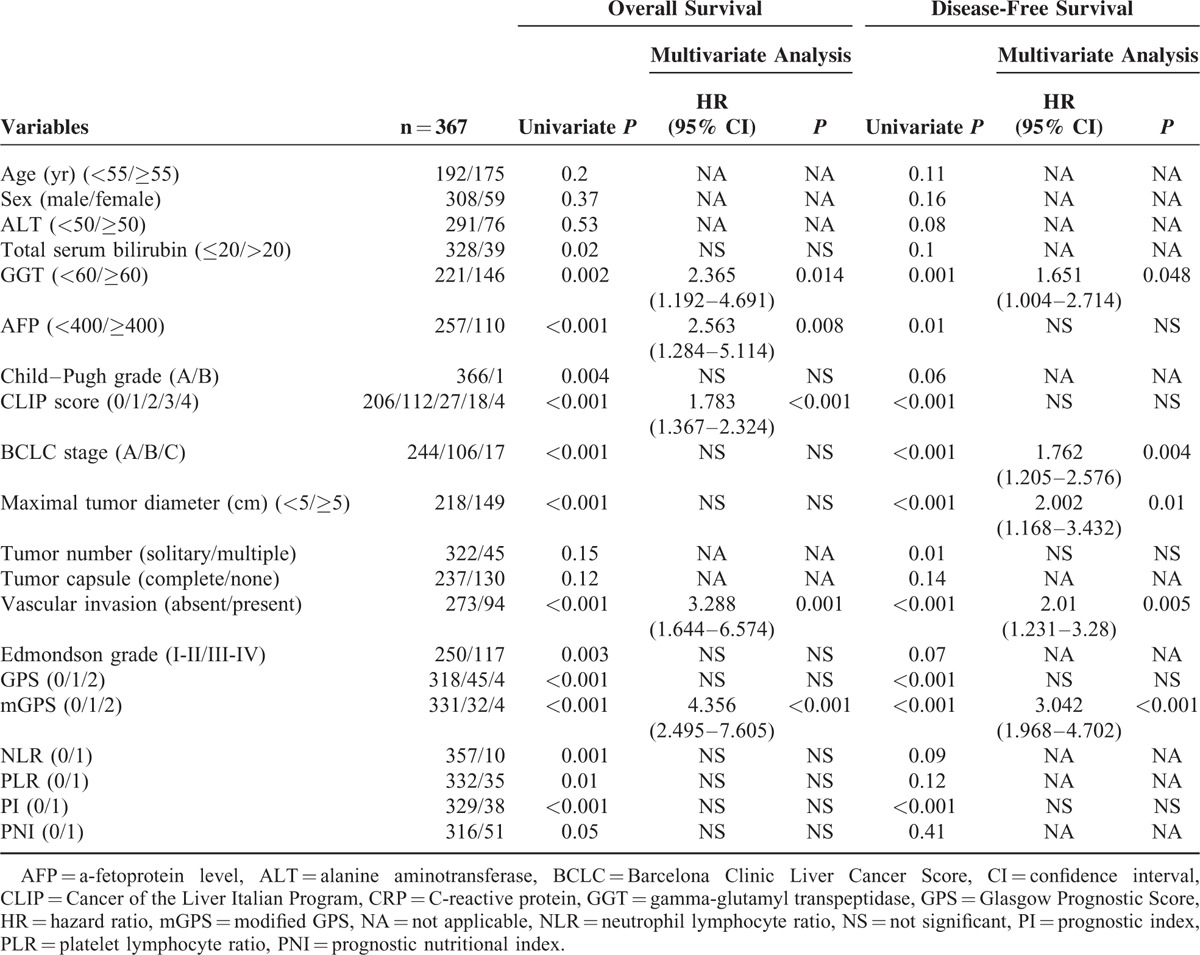
Univariate and Multivariate Analyses of Prognostic Factors in Test Cohort

### Comparative Discriminatory Performance of Staging Systems

The discriminatory capacity of each prognostic system was compared by means of receiver operating characteristics curve analysis. The area under the receiver operating characteristics curve (AUC) value was calculated for each prognostic system, as shown in Table 4. The BCLC stage and CLIP score have superior discriminative abilities as compared with the inflammation-based scores, and the GPS, mGPS, and PI have similar AUC values, which were higher than other inflammation-based prognostic scores.

**TABLE 4:**
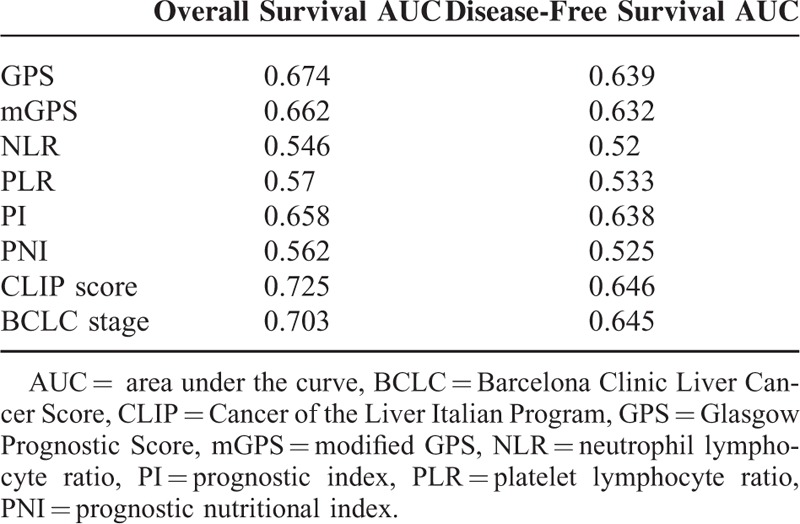
Comparison of the AUC between Inflammatory Prognostic Scores and Staging Systems in Test Cohort

### Validation of Inflammation-Based Prognostic Scores

The inflammation-based prognostic scores were further assessed for their prognostic power and discriminative ability in the validation cohort. In accordance with the association of prognostic scores and clinicopathologic parameters in the test cohort, the GPS, mGPS, and PI remained associated with tumor size, vascular invasion, BCLC stage, and CLIP score (Supplementary Table S2, http://links.lww.com/MD/A406). Univariate and multivariate analyses showed that the mGPS remained a significant predictor of OS and DFS (Supplementary Figures.1 and 2, http://links.lww.com/MD/A406, and Supplementary Table S3, http://links.lww.com/MD/A406). The discriminatory capacity of the BCLC stage and CLIP score, as assessed by the AUC, was superior to the inflammation-based prognostic scores, including the mGPS (Supplementary Table S4, http://links.lww.com/MD/A406).

## DISCUSSION

The host systemic inflammatory response plays an important role in cancer development and progression.^25–27^ The most commonly used biomarker of systemic inflammation is serum CRP, which is produced in the liver. Recent studies have revealed that elevated CRP levels may be associated with tumor size, distant metastasis, and vascular invasion, resulting in poor prognosis in patients with various cancers, including HCC.^6,28^ Furthermore, the presence of an inflammatory response is proposed to be pathogenic in the development of cancer-associated malnutrition and a decline in serum albumin levels is a poor prognostic factor for long-term survival in patients with various cancers.^29,30^ Moreover, several studies have shown that inflammation-based prognostic scores, including a combination of serum CRP and albumin as the GPS or mGPS, are significant prognostic markers of HCC.

In our analysis, the inflammation-based prognostic scores were associated with a number of clinicopathologic characteristics of HCC. Consistent with a study by Huang et al^31^ elevated GPS, mGPS, and PI were associated with factors of tumor progression, such as maximal tumor diameter, vascular invasion, and higher CLIP score and BCLC stage, suggesting that the presence of a systemic inflammatory response is predictive of a more aggressive clinical phenotype; however, our data showed that the majority of elevated inflammation-based prognostic scores were not associated with factors indicating reduced liver function, such as higher total bilirubin, alanine transaminase, GGT, lower albumin, and higher Child–Pugh scores, which is inconsistent with a study by Kinoshita et al.^32^ This discrepancy may be due to the higher proportion of patients with early stage disease and proximate normal liver function of the patients eligible for surgical resection in our study.

To the best of our knowledge, few studies have compared inflammation-based prognostic scores in patients with HCC. Pinato et al^33^ explored the prognostic impact of a panel of inflammatory-based scores, including the mGPS, NLR, and PLR in independent HCC cohorts, and found that the mGPS emerged as independent predictor of OS and the predictive accuracy appeared to be superior to that of the NLR and PLR. Recently, in the Glasgow Inflammation Outcome Study, Proctor et al^12^ reported that the mGPS was a powerful prognostic factor independent of tumor site and was superior to other inflammation-based scores with greater consistency and more general use. The results of our study are consistent with the above observations and further demonstrated that the mGPS appears to be a more important predictor than any other score. In addition, the usefulness of CRP as a prognostic factor of DFS after liver resection for HCC was recently reported.^6^ According to the authors, 75.3% of patients undergoing hepatic resection with preoperative CRP levels of >1.0 mg/dL experienced HCC recurrence after 1 year. Therefore, based on existing validation studies, scores that incorporate CRP and serum albumin (mGPS and GPS) may reflect both the presence of a systemic inflammatory response and a progressive nutritional decline in patients with cancers, and were superior to those based on components of the circulating white cell count (NLR, PLR) or in combination with albumin alone (PNI).Therefore, any further development of inflammation-based prognostic scores to improve the power of prognosis should also include the prototypical acute phase protein CRP.

In the present study, univariate analysis showed that the GPS, mGPS, PLR, and PI were significantly associated with OS in both cohorts, although multivariate analysis showed that only the mGPS was an independent predictor of OS. Further analysis revealed that the GPS and mGPS had similar discriminative abilities and were superior to other inflammation-based scores. Kinoshita et al^32^ reported that the GPS is more suitable than the mGPS with regard to discriminating ability and the monotonicity of gradients. Although the majority of patients enrolled in his study received nonsurgical treatment due to advanced stage disease, poor liver function and reduced albumin may have affected patient outcomes equally. Furthermore, multiple and various treatment sessions were adopted due to tumor recurrence in his study. Consequently, it was difficult to evaluate the prognostic value of the GPS and mGPS accurately. A previous study compared inflammation-based scores, including the GPS and mGPS, in patients undergoing hepatectomy and concluded that the prognostic ability of the GPS was superior to that of other inflammation scores, although in this study, the GPS and mGPS had the same C-index value, which is an assessment of discriminative ability, whereas the AUC, another index to test prognostic power, in the present study had the same result as that of the above-cited studies. Even more importantly, in the present study, the mGPS was an independent predictor of DFS, an important part of prognosis especially for HCC, as compared with other inflammation-based scores, including the GPS. Therefore, we concluded that the mGPS may be more suitable than the GPS for patients with resectable HCC.

We also examined the predictive ability of the BCLC stage and CLIP score to assess survival. The BCLC staging system is the most comprehensive currently available and has been described “as the standard classification that is used for trial design and clinical management of patients with HCC.”^34–36^ In addition, the BCLC staging system was designed with the ability to provide therapeutic options for patients at different stages of disease. The CLIP system is generally accepted as more suitable for predicting survival of HCC patients who receive nonsurgical treatments than the BCLC system. Conversely, in recent studies, the CLIP was shown to have a better discriminatory ability and was reliable for long-term prognostic prediction, independent of the treatment strategy in HCC.^37,38^ In our study, multivariate analysis of the validation cohort showed that the BCLC stage was independently associated with OS and DFS. Although the discriminative abilities are about the same, the BCLC staging system seems to be regarded as a more stable and accurate staging system of HCC.

Despite the BCLC stage and CLIP score having more accurate discriminative ability of prognosis in our study, for a scoring system to be effective, it must be simple and easy to apply in routine clinical practice for prognostication of patients with primary HCC before treatment; however, the mGPS comprises only two sets of routinely available blood tests and utilizes a standardized methodology and thresholds without additional imaging techniques or histologic examinations before commencing treatment. In this regard, the mGPS appears to be more clinically useful. Certainly, together with current tumor-staging methods, such as the BCLC and CLIP staging systems, measures with the mGPS will further provide accurate prediction of treatment outcome, and therefore better treatment allocation in patients with HCC, as reported previously.^32,39^

Although significant differences in OS were found across all staging systems, the curves of OS and DFS for the mGPS and CLIP partially overlapped, suggesting their classification ability may not be perfect, as they were unable to accurately divide the patients into different outcome groups; however, their classification value maybe less than perfect because most patients undergoing hepatic resection have a lower CLIP score (CLIP 0, 417; CLIP 1, 215; CLIP 2, 52) and mGPS (mGPS 0, 652; mGPS 1,64) at an early stage of HCC, which is consistent with the findings of some recent studies that included patients with resectable and unresectable HCC.^19,33,40^ Based on these findings, a new modified GPS is necessary to solve these problems and improve prognostication. Ishizuka et al^39^ assessed and compared the predictive values of a new mGPS, the hepatic GPS, which incorporates a lower CRP cut-off level in patients undergoing surgery for HCC. Their results clearly disclosed that the hepatic GPS has a better predictive value and ability to classify patients than the GPS, mGPS, or even the CLIP score, and is considered to be an important factor predictive of postoperative mortality. A challenge for the future is to develop a validation and consensus score for use as a simple and accurate stratification parameter in HCC. Based on the above findings, we have reason to believe that a creative version of the mGPS with an adequate CRP cut-off value will be a simple and sensitive prognostic scoring system of HCC with classification ability.

The mechanism by which systemic inflammation may impact survival is not completely understood. A number of studies appear to reinforce the biologic plausibility behind systemic inflammation and the prognosis of HCC. It has been proposed that elevated levels of CRP, which is produced by hepatocytes in response to inflammatory cytokines, particularly interleukin-6, indicates T-lymphocyte impairment, which reflects compromised cell-mediated immunity and is associated with poor outcome in malignancy.^41^ On the contrary, CRP levels are directly associated with circulating concentrations of vascular endothelial growth factor, a proangiogenic environment growth factor, allowing unrestrained tumor growth and dissemination. Furthermore, elevated CRP levels and impaired nutritional status have been associated with increased toxicity from chemotherapy.^42^ The activity of cytochrome 3A and cellular response to chemotherapy-induced DNA damage were impaired due to systemic inflammatory responses, which resulted in impaired drug response. Consequently, the mGPS, as a reflection of the systemic inflammatory response and progressive malnutrition, should be incorporated into the planning and monitoring of cancer treatments, such as with HCC patients undergoing transarterial chemoembolization or sorafenib treatment; however, this hypothesis requires further evaluation in a prospectively designed trial.

A number of limitations to the present study should be addressed, such as the retrospective and single-center nature of our study and the median follow-up period of 24 months (range, 3–32 months), which may be insufficient to predict 5-year survival and recurrence; however, recurrence of HCC mostly occurs within 2 years after curative resection, at a rate of up to 62.4% to 77.8%. Also, the data presented here were based on a representative sample of patients who underwent hepatic resection for HCC and have been tested and validated in two large datasets. In addition, to the best of our knowledge, this study is the first to evaluate the association of inflammation-based scores and recurrence in resectable HCC. We believe that cross-validation in independent cohorts in a multicenter or possibly a prospective setting should be further explored in the future.

In conclusion, the results of the present study suggest that the mGPS, an inflammation-based prognostic score, is an independent prognostic marker for poor prognosis in patients with HCC undergoing hepatectomy and is superior to other inflammation-based prognostic scores. In addition, the mGPS is simple to construct from laboratory measurements that are routinely assessed in patients before treatment. Therefore, these findings highlight a potential role for the mGPS in predicting prognosis in patients with HCC.
